# Rand’s Medical Chemistry

**Published:** 1855-05

**Authors:** 


**Rand's Medical Chemistry.**

This little volume, of 250 pages, has been received, and upon examination we find it to be what its author, B. H. Rand, A. M., M. D., Philadelphia, claims for it—an "outline of Medical Chemistry, which outline is to be filled up by aid of text-books or of lectures."

He first treats of the principles of chemistry: proceeds then to to the discussion of Inorganic Chemistry; following this is a chapter on Organic Chemistry, which is valuable to the student in his physiological enquiries and of general reference. Then follows an appendix, embracing a toxicological table, a list of the more common incompatibles, and concludes with a glossary, containing terms and synonyms, making plain what are often to students very difficult to comprehend.

Beginners would find this *multum in parvo* offering a highly useful and valuable auxillary in the progress of their studies, and we take this occasion to recommend it to their considerate attention.

It is very neatly executed, and published by Lindsay & Blackiston, Philadelphia.

